# Outstanding Reviewers for *Nanoscale Advances* in 2023

**DOI:** 10.1039/d4na90067g

**Published:** 2024-07-01

## Abstract

We would like to take this opportunity to thank all of *Nanoscale Advances*' reviewers for helping to preserve quality and integrity in chemical science literature. We would also like to highlight the Outstanding Reviewers for *Nanoscale Advances* in 2023.

## Abstract 2

We would like to take this opportunity to highlight the Outstanding Reviewers for *Nanoscale Advances* in 2023, as selected by the editorial team for their significant contribution to the journal.

## Graphical abstract

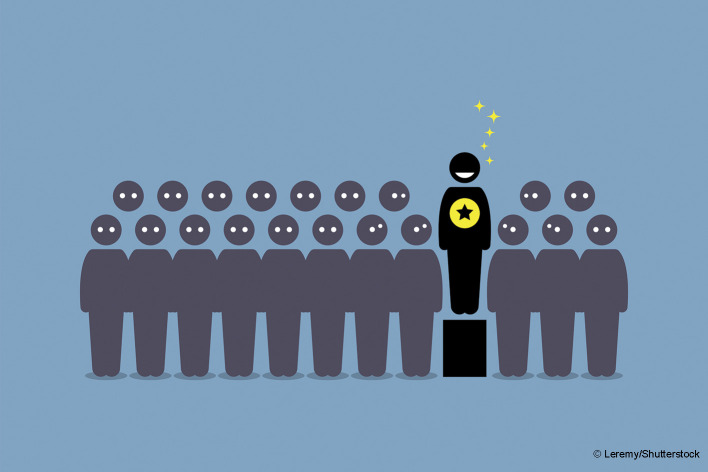

Each one of our outstanding peer reviewers has been carefully selected by our editorial team and includes active researchers who have made significant contributions to peer review and have gone above and beyond in their actions.

We announce our Outstanding Reviewers annually and each receives a certificate to give recognition for their contribution. The reviewers have been chosen based on the number, timeliness and quality of the reports completed during 2023.

“*Keep up the good work. Outstanding Reviewer Awards are given by the editors of Nanoscale Advances to scientists who provided constructive, benevolent, and punctual reviews during the previous year. I personally want to add my thanks to these Outstanding Reviewers and also thank everyone who has reviewed manuscripts for Nanoscale Advances.*” – Professor Dirk Guldi, Editor-in-Chief.

“*High-quality peer-review, to a great extent, bridges the gap between initial manuscript level and journal's publication standard, paving the way towards better content presentation and fresh knowledge spread for world-wide research communities. Therefore, we're very delighted to recognize the Nanoscale Advances Outstanding Reviewers who have devoted continuous effort on manuscript review. Without their excellent professionalism and dedicated attitude, this journal's publications cannot be elevated. With concerted effort from all sides, we're bound to make greater progress and generate more novel academic ideas on this promising open-access platform.*” – Professor Yue Zhang, Editor-in-chief

 

Dr Nikhil Bhalla

Ulster University

ORCID: 0000-0002-4720-3679

 

Dr Minju Kim

National University of Singapore

ORCID: 0000-0002-0962-2661

 

Dr Bo Li

Kennesaw State University

ORCID: 0000-0001-9407-9503

 

Professor Junsuk Rho

Pohang University of Science and Technology

ORCID: 0000-0002-2179-2890

 

Professor Hui Wang

University of South Carolina

ORCID: 0000-0002-1874-5137

 

Dr Hui Xu

Nanjing Tech University

ORCID: 0000-0002-9232-9625

 

We would also like to thank the *Nanoscale Advances* Editorial Board and Advisory Board and the nanoscience community for their continued support of the journal, as authors, reviewers and readers.

We continue to work on improving the diversity of our reviewer pool to reflect the diversity of the communities that we serve.

 

Dirk Guldi, Editor-in-chief

Yue Zhang, Editor-in-chief

Jeremy Allen, Executive Editor

